# Synthesis of Stable and Soluble One-Handed Helical Homopoly(substituted acetylene)s without the Coexistence of Any Other Chiral Moieties via Two-Step Polymer Reactions in Membrane State: Molecular Design of the Starting Monomer

**DOI:** 10.3390/molecules17010433

**Published:** 2012-01-04

**Authors:** Yunosuke Abe, Toshiki Aoki, Hongge Jia, Shingo Hadano, Takeshi Namikoshi, Yuriko Kakihana, Lijia Liu, Yu Zang, Masahiro Teraguchi, Takashi Kaneko

**Affiliations:** 1 Graduate School of Science and Technology, Niigata University, Ikarashi 2-8050, Nishi-Ku, Niigata 950-2181, Japan; 2 Department of Polymeric Material and Engineering, Qiqihar University, Wenhua street 42, Qiqihar 161006, China; Email: jiahongge11@hotmail.com; 3 Chemical Resources Laboratory, Tokyo Institute of Technology, 4259 Nagatsuta, Midori-ku, Yokohama 226-8503, Japan; Email: hadano.s.aa@m.titech.ac.jp; 4 Material Science and Engineering, Kitami Institute of Technology, 165 Koen-cho, Kitami, Hokkaido 090-8507, Japan; Email: takenami@mail.kitami-it.ac.jp

**Keywords:** one-handed helicity, chirality, polymer reaction, membrane, phenylacetylene, asymmetric polymerization, solubility

## Abstract

A soluble and stable one-handed helical poly(substituted phenylacetylene) without the coexistence of any other chiral moieties was successfully synthesized by asymmetric-induced polymerization of a chiral monomer followed by two-step polymer reactions in membrane state: (1) removing the chiral groups (*desubstitution*); and (2) introduction of achiral long alkyl groups at the same position as the *desubstitution* to enhance the solubility of the resulting one-handed helical polymer (*resubstitution*). The starting chiral monomer should have four characteristic substituents: (i) a chiral group bonded to an easily hydrolyzed spacer group; (ii) two hydroxyl groups; (iii) a long rigid hydrophobic spacer between the chiral group and the polymerizing group; (iv) a long achiral group near the chiral group. As spacer group a carbonate ester was selected. The two hydroxyl groups formed intramolecular hydrogen bonds stabilizing a one-handed helical structure in solution before and after the two-step polymer reactions in membrane state. The rigid long hydrophobic spacer, a phenylethynylphenyl group, enhanced the solubility of the starting polymer, and realized effective chiral induction from the chiral side groups to the main chain in the asymmetric-induced polymerization. The long alkyl group near the chiral group avoided shrinkage of the membrane and kept the reactivity of *resubstitution* in membrane state after removing the chiral groups. The g value (g = ([θ]/3,300)/ε) for the CD signal assigned to the main chain in the obtained final polymer was almost the same as that of the starting polymer in spite of the absence of any other chiral moieties. Moreover, since the one-handed helical structure was maintained by the intramolecular hydrogen bonds in a solution, direct observation of the one-handed helicity of the final homopolymer has been realized in CD for the solution for the first time.

## 1. Introduction

*Conjugated* polymers like polyacetylenes have aroused interest because of their unique properties such as optical resolution and enantioselective recognition [1,2,3]. Many kinds of polymers having chiral structures in their main chains and/or pendant groups have been synthesized. Among them, *soluble* chiral polymers whose chiral structures arise *solely from one-handed helical conformations of the conjugated main chains* have so far only been synthesized by the helix-sense-selective polymerization (**HSSP**) of an achiral phenylacetylene monomer by using a chiral catalytic system developed in our laboratory [4,5,6,7,8,9,10,11,12,13,14,15].

In general, there are two methods to synthesize *soluble* chiral polymers whose chiral structures are present alone in the main chain as asymmetric carbons and /or as a one-handed helical conformation. One is **HSSP** [16,17,18,19] of an achiral monomer by using a chiral catalyst as a chiral source and the other is asymmetric-induced polymerization (**AIP**), by using achiral catalysts, of monomers having a chiral group as a chiral source, followed by removing the chiral groups by a polymer reaction of the resulting polymer *in solution* (**AIP-R**) [20,21,22,23,24,25,26,27,28,29]. However, the examples where the two methods have been applied to synthesize *conjugated* polymers are very few. In fact, there has been only one example using **HSSP** reported by our group [4,5,6,7,8,9,10,11,12,13,14,15] and no examples of the use of **AIP-R** for obtaining *soluble* chiral *conjugated* polymers.

Recently, we have successfully synthesized *soluble* chiral *co*poly(substituted phenylacetylene)s whose chiral structures are only in one-handed helical conformations by asymmetric-induced *co*polymerization of a chiral monomer with an achiral *co*monomer having a hydrophobic group(**AIP**) and subsequent *desubstitution* of the chiral pendant groups from the resulting polymers by one-step polymer reaction in membrane state (**RIM**; in the case of the same polymer reaction in solution, the one-handed helicity was racemized during the reaction [30]). We also found the efficiency in the asymmetric induction to the main chain in this two-step method (**AIP-RIM**) was better than that of **HSSP**. However, the *homo*polymer by **AIP** of the chiral monomer was insoluble and therefore chiralities could not be measured directly in CD for the solutions.

**Scheme 1:**
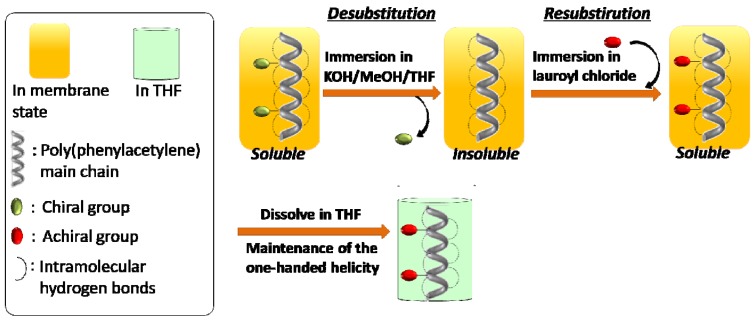
Two-step synthetic route to stable and soluble one-handed helical homo- poly(substituted acetylene)s without the coexistence of any other chiral moieties.

**Figure 1:**
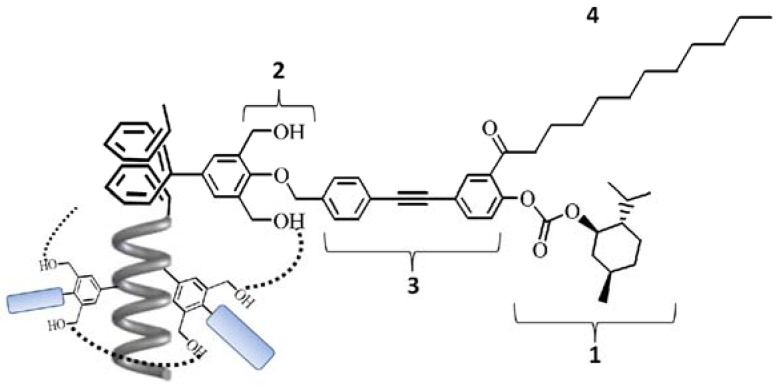
Molecular design of the starting one-handed helical polymer in this study. (**1**) Removable chiral source by hydrolysis for *desubstitution*; (**2**) Stabilization of the one-handed helical backbone by the intramolecular hydrogen bonds; (**3**) To enhance solubility of the polymer by the hydrophobicity. Effective chiral induction to the backbone from the chiral groups by the rigidity; (**4**) To enhance reactivity for *resubstitution* by enhancing the reaction space.

**Scheme 2:**
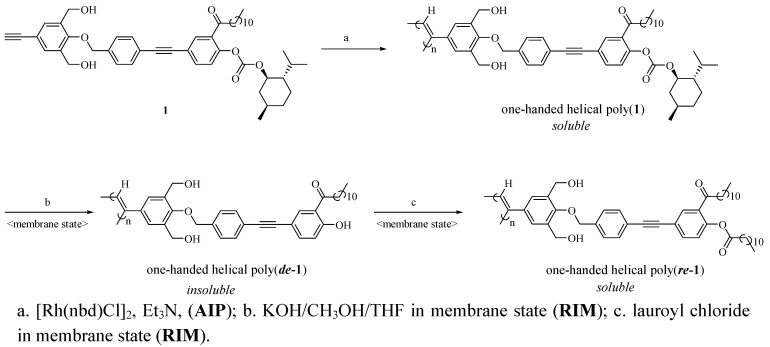
Synthesis of soluble one-handed helical poly(***re*-1**) by asymmetric-induced polymerization followed by polymer reactions in membrane state (**AIP-RIM**).

In this study, a *solublehomo*polymer having its chirality only in the one-handed helical backbone was synthesized by **AIP-RIM** (Scheme 1) of a chiral phenylacetylene monomer (**1**) (Figure 1 and Scheme 2)*.*

This method consisted of **AIP** of **1** and two-step polymer reactions (*desubstitution and resubstitution*) in membrane state (**RIM**): (1) removing the chiral groups while maintaining the one-handed helical backbone (*desubstitution*); and (2) introduction of achiral long alkyl groups at the same position to enhance solubility of the resulting polymer while maintaining the one-handed helical backbone (*resubstitution*). As a result, CD for the resulting polymer could be measured in solution. To realize this purpose, the monomer **1** was designed precisely as follows (Figure 1 and Scheme 2): (i) a chiral group bonded by a group easily hydrolyzed; (ii) two hydroxyl groups to stabilize the one-handed helical backbone of the resulting polymer by the intramolecular hydrogen bonds; (iii) a phenylethynylphenyl spacer to enhance the solubility of the polymers, and to realize effective chiral induction from the chiral side group to the main chain in **AIP** [31]; (iv) a long achiral alkyl group near the chiral group to avoid shrinkage of the polymer membrane and the reactivity of *resubstitution* in **RIM** after removing the chiral groups.

## 2. Results and Discussion

### 2.1. Synthesis of a Soluble One-Handed Helical Poly(**1**) Having Chiral Pendant Groups by Asymmetric-Induced Polymerization(**AIP**) of **1**

**Figure 2:**
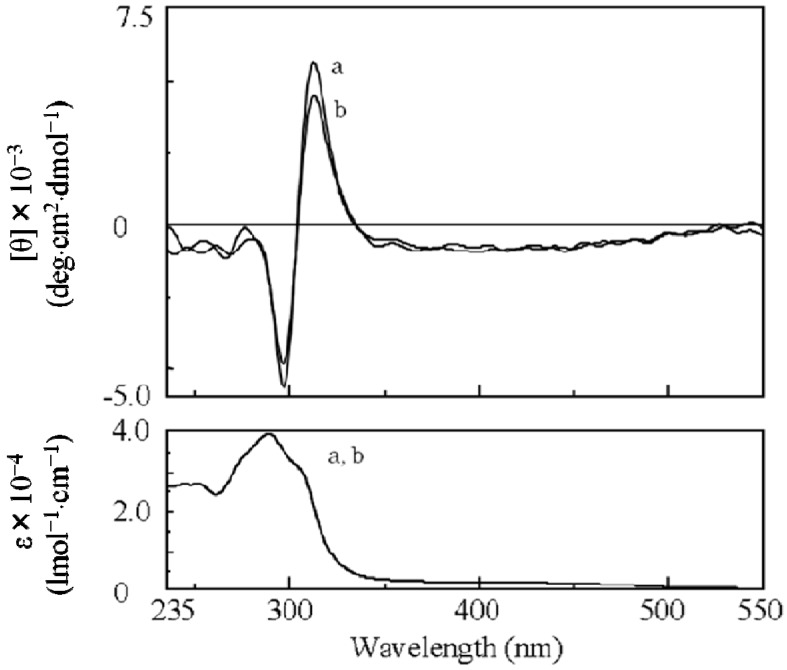
CD and UV-Vis spectra of poly(**1**) at (a) 25 °C and (b) 60 °C in THF.

**Figure 3:**
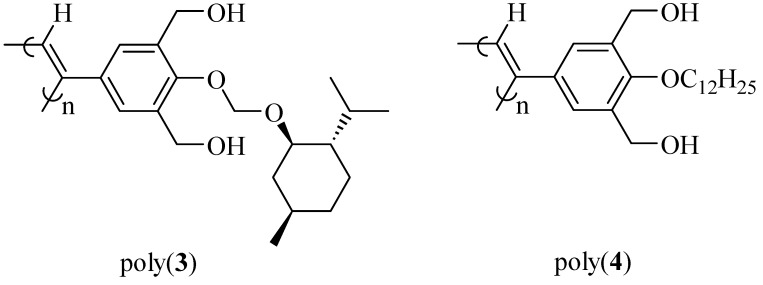
Chemical structures of poly(**3**) and poly(**4**).

**Figure 4:**
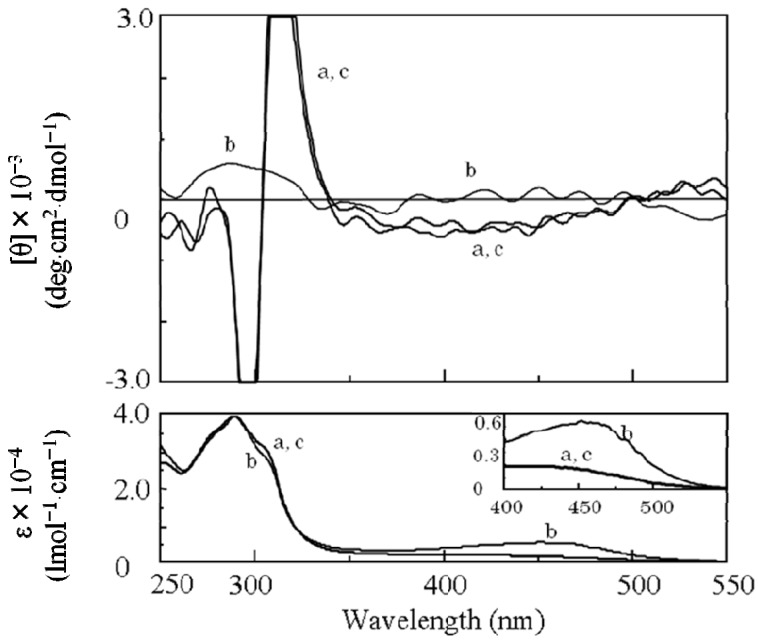
CD and UV-Vis spectra of poly(**1**) in (a) THF, (b) THF/DMSO = 80/20 (v/v) and (c) THF/DMSO = 99/1 (v/v) ((c) was the solution prepared by addition of THF to the solution of (b)).

By introduction of the hydrophobic phenylethynylphenyl spacer, a soluble poly(**1**) was successfully synthesized by **AIP** (Scheme 2 and Table 1) [32]. The value of the specific rotation of poly(**1**) was much higher than that of the corresponding monomer (Table 1). In addition, since CD signals assigned to the main chain were observed for poly(**1**), chiral induction to the main chain was confirmed (Figure 2a). In spite of the long distance of the spacer (23 Å), effective chiral induction from the chiral group to the main chain was observed in **AIP** owing to rigidity of the spacer [31,33]. Moreover, one-handed helicity of poly(**1**) was stable when it was heated at 60 °C (Figure 2b). Since the CD and UV spectra for poly(**1**) (Figure 2a) were very similar to those for a one-handed helical poly(substituted phenylacetylene) (poly(**4**) in Figure 3) stabilized by intramolecular hydrogen bonds we previously reported [4], the backbone of poly(**1**) is also thought to be kept by intramolecular hydrogen bonds. When DMSO as a polar solvent was added to the THF solution of poly(**1**) showing CD around 450 nm assigned to the main chain (Figure 4a), the CD band disappeared and the UV band shifted to longer wavelengths (Figure 4b). The finding was thought to show that the intramolecular hydrogen bonds were broken, the helical pitch of the conjugated conformation was extended, and then the one-handed helical conformation was racemized. At the same time, a new CD signal assigned to the chiral group in the monomer unit appeared at 250 to 350 nm (Figure 4b).

**Table 1:** Characterizations of **1**, poly(**1**) ^a^ and poly(***re*-1**).

Code	Yield^ b^/%	*M*_w_^c^/×10^5^	*M*_w_/*M*_n_^c^	[α]_D_^20^^ d^/°	g_450_^e^/×10^−7^
**1**	-	-	-	−18.4	0
poly(**1**)	98.6	27	16	−176	2.7
poly(*re*-**1**)	91.4	5.1	10	117	2.5

^a^ At room temperature in toluene, [**1**] = 0.1 mol/l, [**1**]/ [[Rh(nbd)Cl]_2_] = 100; ^b^ methanol insoluble part; ^c^ by GPC; ^d^in THF; ^e^ g = ([θ]/3300)/ε.

### 2.2. Synthesis of an Insoluble One-Handed Helical Poly(**de-1**) Having no Chiral Pendant Groups by Removing the Chiral Groups (Desubstitution) Quantitatively from Poly(**1**) in Membrane State (**RIM**)

**Figure 5:**
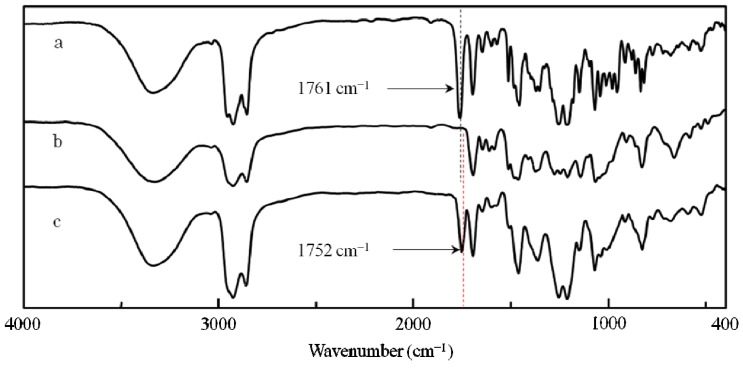
IR spectra of (a) poly(**1**), (b) poly(***de*-1**) and (c) poly(***re*-1**) (film).

**Figure 6:**
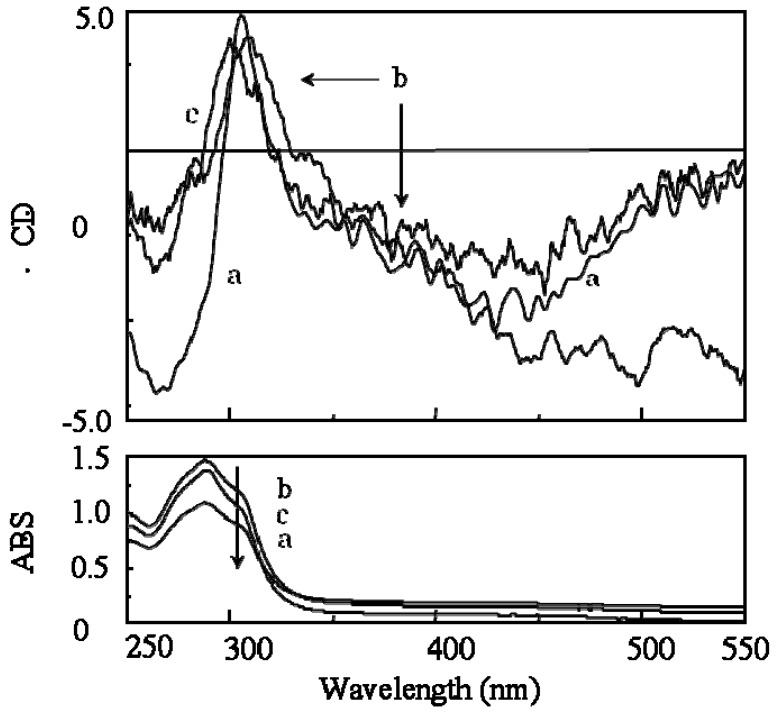
CD and UV-Vis spectra of (a) poly(**1**), (b) poly(***de*-1**) and (c) poly(***re*-1**) in membrane state.

After the desubstitution reaction of the chiral groups from poly(**1**) in membrane state (**RIM**), the obtained poly(**de-1**) became insoluble in any organic solvents. Therefore, the conversion of **RIM** was estimated by IR and loss of the weight of the membrane. Since the absorption at 1761 cm^−1^ assigned to the carbonyl stretching vibration in the ester carbonate bonded with the chiral groups completely disappeared (Figure 5a,b) and the actual weight loss was almost the same as the theoretical value (99.7%), quantitative removal of the chiral groups was confirmed. Because the CD and UV-Vis spectra of poly(**de-1**) were almost the same as those of poly(**1**) in membrane state (Figure 6a,b), it was suggested that poly(**de-1**) maintained the one-handed helical structure of the original polymer in membrane state during **RIM**. Since the broad absorption band for the hydroxyl groups in poly(**de-1**) at 3750 to 3100 cm^−1^ was observed similar to that in poly(**1**) (Figure 5a,b), the one-handed helicity may be maintained by the intramolecular hydrogen bonds during the polymer reaction in membrane state.

### 2.3. Synthesis of a Soluble One-Handed Helical poly(***re*-1**) Having no Chiral Pendant Groups by Quantitative Introduction of Achiral Groups (Resubstitution) to Poly(***de*-1**) in Membrane State(**RIM**)

#### 2.3.1. Quantitative Introduction of Achiral Groups (*Resubstitution*) by **RIM**

**Figure 7:**
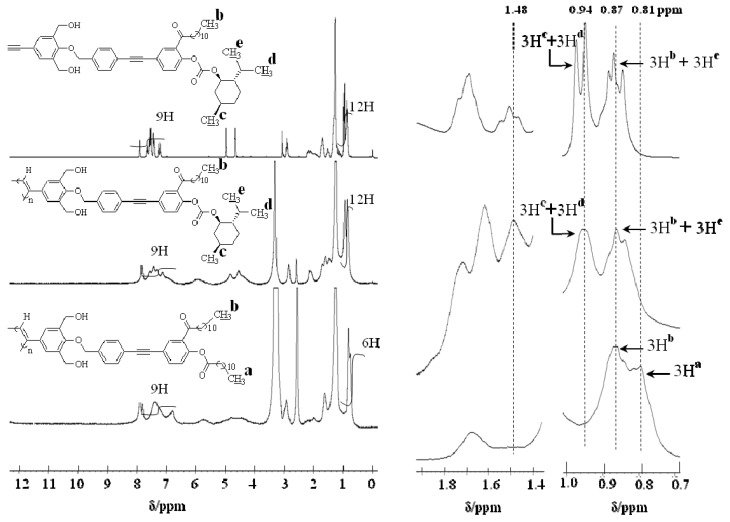
^1^H-NMR spectra of **1** in CDCl_3_ and poly(**1**) and poly(***re*-1**) in CDCl_3_/DMSO-*d*_6_.

After introduction (*resubstitution*) reaction of the achiral groups by the reaction of phenolic alcohol in poly (***de*-1**) with lauroyl chloride in membrane state (**RIM**), unlike poly(***de*-1**) the obtained poly(***re*-1**) became soluble in chloroform and THF. It was found that the reaction was almost quantitative by IR and ^1^H-NMR. The ^1^H-NMR spectra of **1**, poly(**1**) and poly(***re*-1**) are shown in Figure 7 (no data is available for poly(***de*-1**) because of its insolubility). The signals in the chiral menthoxy group of poly(**1**) observed at 1.48 and 0.94 ppm disappeared completely in poly(***re*-1**), indicating the absence of the chiral groups in poly(***re*-1**). In addition, since the value of the ratio of the integral values of the two methyl protons in the two undecyl groups at 0.87 and 0.81 ppm to the nine aromatic protons assigned to three types of the benzene rings at 7.80–6.73 ppm was the same as theoretical one(6:9), the quantitative *de-* and* re-substitution* were confirmed. It was also confirmed by the fact that the absorption at 1752 cm^−1^in the IR spectra assigned to the carbonyl stretching vibration of the phenyl ester group newly appeared (Figure 5b,c). Since the broad absorption for the hydroxyl groups in poly(***re-*1**) at 3750 to 3100 cm^−1^ was observed similar to that in poly(**1**) and poly(***de*-1**) (Figure 5a–c), the one-handed helicity was thought to be maintained by the hydrogen bonds during the polymer reaction in membrane state.

**Scheme 3:**
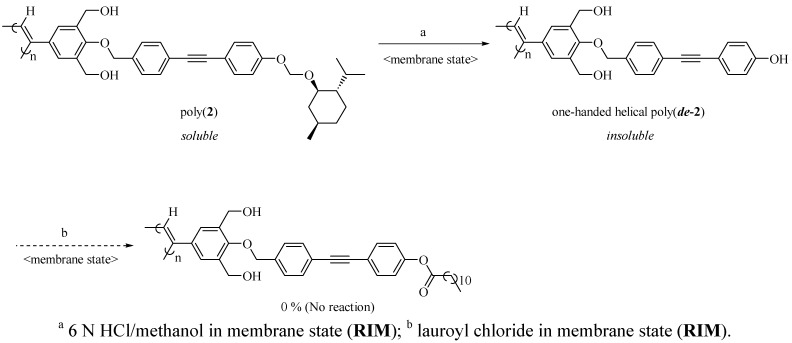
Synthesis of insoluble one-handed helical poly(***de*-2**) by polymer reaction in membrane state(**AIP-RIM**).

In order to investigate the effect of the long alkyl group near the *resubstitution* reaction site on the reactivity, a chiral monomer(**2**) which has no such alkyl groups was synthesized and polymerized as shown in Scheme 3. **AIP-RIM** of **2** gave poly(***de*-2**), and *resubstitution* in poly(***de*-2**) membranes was attempted. As a result, the *resubstitution* did not proceed in poly(***de*-2**) membranes. In the case of the absence of the long alkyl group, shrinkage of the membrane and decrease of the reaction space for *resubstitution* occurred after removing the bulky menthoxy groups by **RIM**. In fact, the ratio of decrease (%) of the diameter from poly(**2**) to poly(***de*-2**) membranes was 13% which was larger than that from poly(**1**) to poly(***de*-1**) membrane (4%). These results indicated that introduction of the long alkyl group near the chiral group reduced shrinkage of the membrane and kept the reactivity of *resubstitution* in membrane state of poly(***de*-1**) after removing the chiral groups.

#### 2.3.2. Evidence of the One-Handed Helicity of Poly(***re*-1**) Prepared by Two-Step **RIM**

**Figure 8:**
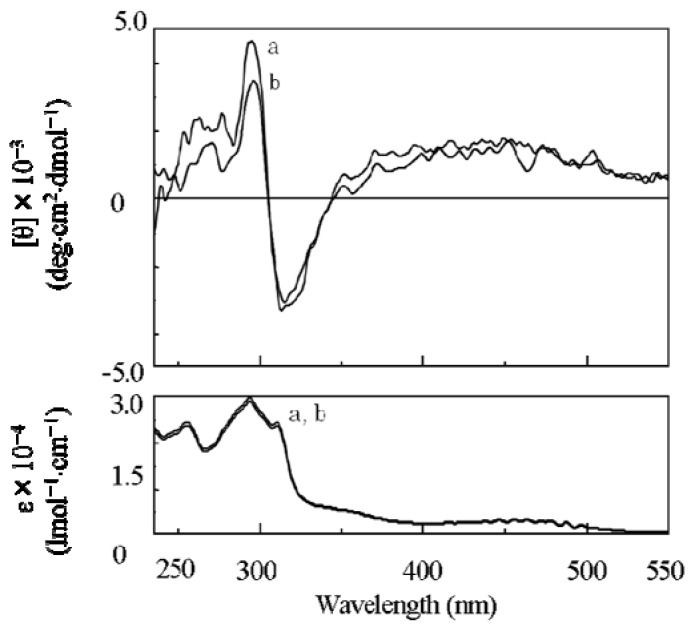
CD and UV-Vis spectra of poly(***re*-1**) at (a) 20 °C and (b) 60°C in THF.

**Figure 9:**
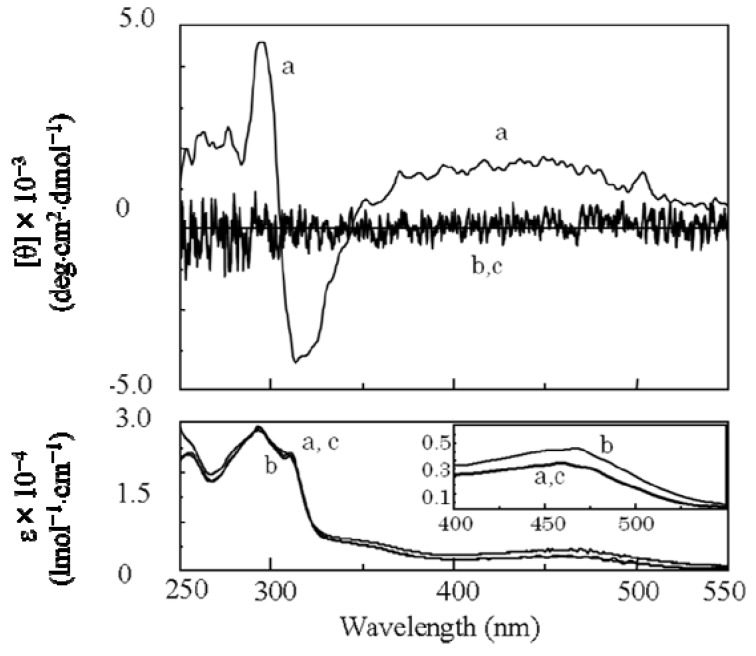
CD and UV-Vis spectra of poly(***re*-1**) in (a) THF, (b) THF/DMSO= 80/20(v/v) and (c) THF/DMSO = 99/1 (v/v) ((c) was the solution prepared by addition of THF to the solution of b).

Since the obtained soluble poly(***re*-1**) showed CD signals assigned to the main chain in THF (Figure 8), finally by the two-step **RIM**s we obtained a one-handed helical homopolymer without any other chiral moieties which has the same structure of the backbone as that of poly(**4)** (Figure 3) prepared only by **HSSP** of an achiral monomer(**4**). In addition, the g value (=([θ]/3,300)/ε) of the main chain of poly(***re*-1**) was almost the same as that of the starting polymer, poly(**1)** (Table 1). Like poly(**4**), almost no change in the CD intensity of poly(***re*-1**) was observed after heating the solution at 60 °C (Figure 8b). This may be because the one-handed helical structure was stabilized by the intramolecular hydrogen bonds. When DMSO as a polar solvent was added to the THF solution of poly(***re*-1**) showing CD around 450 nm assigned to the main chain (Figure 9a), the CD disappeared and the UV band shifted to longer wavelengths in THF/DMSO [80/20(v/v); Figure 9b], because the intramolecular hydrogen bonds were broken, the helical pitch of the conjugated main chain was extended, and the one-handed conformation was racemized similar to those in poly(**4**). When to the solution of THF/DMSO [80/20(v/v)] where no CD was observed, THF was added to reform intramolecular hydrogen bonds, the resulting solution of THF/DMSO (99/1(v/v)) showed no CD signals (Figure 9c) although hydrogen bonds were recovered. It may be because poly(***re*-1**) took a thermodynamically stable racemic helical structure during reconstruction of intramolecular hydrogen bonds due to the absence of the chiral groups in the side chains.

In summary, we obtained soluble and stable one-handed helical homopolymer without any other chiral moieties which had a main-chain structure similar to that of poly(**4**) previously prepared only by **HSSP** of an achiral monomer(**4**). Therefore, **AIP** followed by the 2-steps **RIM** (**AIP-RIM**) method is equivalent to **HSSP**.

## 3. Experimental

### 3.1. General

All of the solvents used for synthesis and polymerizations of the monomers were distilled as usual. The polymerization initiator, [Rh(nbd)Cl]_2_ (nbd = 2,5-norbornadiene), purchased from Aldrich Chemical was used as received. Average molecular weights (*M*_n_ and *M*_w_) were estimated by gel permeation chromatography (THF as an eluent, polystyrene calibration) using JASCO Liquid chromatograph instruments with PU-2080, DG-2080-53, CO-2060, UV-2070, CD-2095, and two polystyrene gel columns (Shodex KF-807L). NMR spectra were recorded on a JEOL GSX-270 at 270 MHz for ^1^H and 67.5 MHz for ^13^C. IR spectra were recorded on a JASCO FTIR-4200 spectrometer. CD spectra were measured with a JASCO J-720 spectropolarimeter. Specific rotations were recorded with a HORIBA SEPA-200.

### 3.2. Monomer Synthesis (see Scheme 4)

**Scheme 4:**
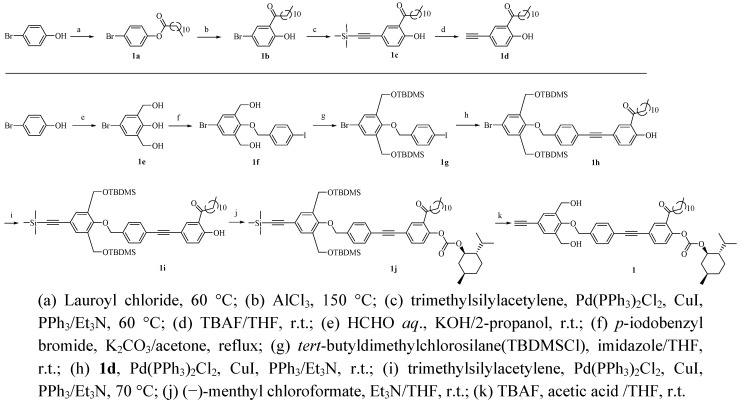
Synthesis of chiral monomer **1**.

#### 3.1.1. Synthesis of 4-Bromophenyl dodecanoate (**1a**)

In a 500 mL three-necked flask, *p*-bromophenol (7.40 g, 42.8 mmol) was heated at 90 °C. After the *p*-bromophenol melted, lauroyl chloride (10.0 mL, 43.2 mmol) was added. The solution was stirred for 2 h at 60 °C. After cooling to room temperature, hexane (200 mL) was added. The solution was washed with aqueous solution of 0.5 wt% NaHCO_3_. The organic layer was dried with anhydrous magnesium sulfate. After filtration, the solvent was evaporated and the crude product was purified by recrystallization (hexane) to give **1a** as a white solid. Yield: 95.6% (14.5 g). ^1^H-NMR (270 MHz, CDCl_3_, ppm) δ: 7.86 (d, 2H, Br-PhH), 7.68 [d, 2H, HPhO(C=O)], 2.54 [t, 2H, PhO(C=O)CH_2_CH_2_], 1.73 (m, 2H, CH_2_CH_2_CH_2_), 1.27 [m, 16H, (CH_2_)_8_ in dodecanone group], 0.88 (t, 3H, CH_2_CH_3_).

#### 3.1.2. Synthesis of 5-Bromo-2-hydroxydodecanophenone (**1b**)

Anhydrous aluminium chloride (3.97 g, 29.8 mmol) was added to **1a** (7.05 g, 19.8 mmol) at 110 °C. The resulting mixture was stirred for 2.5 h at 150 °C. After cooling to room temperature, 1 N HCl was added at 0 °C. After extraction with ether, the organic layer was washed with brine and dried with anhydrous magnesium sulfate. After filtration, the solvent was evaporated and the crude product was purified by silica-gel column chromatography (hexane) to give **1b** as a slightly yellow solid. Yield: 15.9% (1.12 g). Rf: 0.18 (hexane). ^1^H-NMR (270 MHz, CDCl_3_, ppm) δ: 12.3 (s, 1H, PhOH), 7.86 [s, 1H, HPh(C=O)], 7.53 (d, 1H, Br-PhH), 6.83 (d, 1H, HO-PhH), 2.95 [t, 2H, Ph(C=O)CH_2_CH_2_], 1.75 (m, 2H, CH_2_CH_2_CH_2_), 1.27 [m, 16H, (CH_2_)_8_ in dodecanone group], 0.88 (t, 3H, CH_2_CH_3_).

#### 3.1.3. Synthesis of 2-Hydroxy-5-trimethylsilylethynyldodecanophenone (**1c**)

Trimethylsilylacetylene (0.600 mL, 4.22 mmol) was added with a syringe to a mixture of **1b** (1.00 g, 2.84 mmol), triphenylphosphine (59.0 mg, 0.225 mmol), copper iodide (64.3 mg, 0.338 mmol) and bis(triphenylphosphine)palladium (II) chloride (39.5 mg, 56.3 μmol) in triethylamine (19 mL). The mixture was stirred at 60 °C overnight. The formed salt was removed by filtration and the solution was concentrated. After addition of ethyl acetate (100 mL), the resulting solution was washed with brine. The organic layer was dried with anhydrous magnesium sulfate. After filtration, the solvent was evaporated and the crude product was purified by silica-gel column chromatography (hexane) to give **1c** as a slightly viscous liquid. Yield: 86.9% (991 mg). Rf: 0.10 (hexane). ^1^H-NMR (270 MHz, CDCl_3_, ppm) δ: 12.5 (s, 1H, PhOH), 7.89 [s, 1H, HPh(C=O)], 7.53 (d, 1H, Si-≡-PhH), 6.91 (d, 1H, HO-PhH), 2.98 [t, 2H, Ph(C=O)CH_2_CH_2_], 1.73 (m, 2H, CH_2_CH_2_CH_2_), 1.27 [m, 16H, (CH_2_)_8_ in dodecanone group], 0.87 (t, 3H, CH_2_CH_3_), 0.26 [s, 9H, Si(CH_3_)_3_].

#### 3.1.4. Synthesis of 5-Ethynyl-2-hydroxydodecanophenone (**1d**)

Tetrabutylammonium fluoride (1 M in THF) (2.68 mL, 2.68 mmol) was added to a solution of **1c** (991 mg, 2.44 mmol) in THF (7.1 mL). The solution was stirred at room temperature for 6 h. After addition of ethyl acetate, the solution was washed with brine and dried with anhydrous magnesium sulfate. After filtration, the solvent was evaporated and the crude product was purified by silica-gel column chromatography (hexane-ethyl acetate = 20:1) to give **1d** as a white solid. Yield: 92.7% (685 mg). Rf: 0.43 (hexane-ethyl acetate = 20:1). IR (KBr): 3700–3000 (OH), 3312 (≡C-H), 3000–2800 (CH), 1690 (C=O) cm^−1^. ^1^H-NMR (270 MHz, CDCl_3_, ppm) δ: 12.5 (s, 1H, PhOH), 7.93 [s, 1H, HPh(C=O)], 7.56 (d, 1H, ≡‒PhH), 6.95 (d, 1H, HO-PhH), 3.02 (s, 1H, HC≡C), 2.98 [t, 2H, Ph(C=O)CH_2_CH_2_], 1.74 (m, 2H, CH_2_CH_2_CH_2_), 1.27 [m, 16H, (CH_2_)_8_ in dodecanone group], 0.88 (t, 3H, CH_2_CH_3_).

#### 3.1.5. Synthesis of 4-Bromo-2,6-bis(hydroxymethyl)phenol (**1e**)

Compound **1e** was prepared according to our previous method [4].

#### 3.1.6. Synthesis of 1,3-Bis(hydroxymethyl)-2-(4'-iodobenzyloxy)-5-bromobenzene (**1f**)

To a solution of **1e** (4.77 g, 20.5 mmol) and potassium carbonate (4.52 g, 32.7 mmol) in acetone (40 mL), 4-iodobenzyl bromide (7.29 g, 24.5 mmol) was added under reflux. The solution was stirred under reflux for 24 h. The formed salt was removed by filtration and the solution was concentrated. After addition of ethyl acetate (100 mL), the resulting solution was washed with brine. The organic layer was dried with anhydrous magnesium sulfate. After filtration, the solvent was evaporated and the crude product was purified by silica-gel column chromatography (hexane-ethyl acetate = 3:1) to give**1f** as a white solid. Yield: 70.1% (4.80 g). Rf: 0.33 (hexane-ethyl acetate = 3:1). IR (KBr): 3770–3080 (OH), 3082–2800 (CH) cm^−1^. ^1^H-NMR (270 MHz, CDCl_3_, ppm) δ: 7.77 (d, 2H, I-PhH), 7.47 (s, 2H, Br-PhH), 7.25 (d, 2H, PhOCH_2_-PhH), 5.27 (s, 2H, PhOCH_2_Ph), 4.80 [t, 2H, 2(CH_2_OH)], 4.50 [d, 4H, (CH_2_OH)].

#### 3.1.7. Synthesis of 1,3-Bis(*tert*-butyldimethylsilyloxymethyl)-2-(4'-iodobenzyloxy)-5-bromobenzene (**1g**)

*tert*-Butyldimethylchlorosilane (2.21 g, 14.7 mmol) was added to a solution of **1f** (3.00 g, 6.69 mmol) and imidazole (1.82 g, 26.7 mmol) in THF (27 mL) at 0 °C. The solution was stirred for 2 h at room temperature. After addition of ethyl acetate (100 mL), the solution was washed with brine. The organic layer was dried with anhydrous sodium sulfate for 12 h. After filtration, the solvent was evaporated and the crude product was purified by silica-gel column chromatography (hexane) to give **1g** as a white solid. Yield: 89.0% (4.03 g). Rf: 0.53 (hexane-ethyl acetate = 20:1). IR (KBr): 3000–2800 (CH), 1255 (SiC-H), 838 (Si-CH) cm^−1^. ^1^H-NMR (270MHz, CDCl_3_, ppm) δ: 7.72 (d, 2H, I-PhH), 7.49 (s, 2H, Br-PhH), 7.14 (d, 2H, PhOCH_2_-PhH), 4.82 (s, 2H, PhOCH_2_Ph), 4.63 [s, 4H, 2(PhCH_2_OSi)], 0.92 [s, 18H, 2 (Si(CH_3_)_2_C(CH_3_)_3_)], 0.08 [s, 12H, 2(Si(CH_3_)_2_C(CH_3_)_3_)].

#### 3.1.8. Synthesis of 1,3-Bis(*tert*-butyldimethylsilyloxymethyl)-2-{4'-[4''-hydroxy-3''-(1-oxododecyl)phenyl-ethynyl]benzyloxy}-5-bromobenzene (**1h**)

To a mixture of **1g** (1.61 g, 2.38 mmol), triphenylphosphine (7.13 mg, 27.2 μmol), copper iodide (7.76 mg, 40.8 μmol) and bis(triphenylphosphine)palladium (II) chloride (4.77 mg, 6.80 μmol) in triethylamine (11.3 mL), **1d**(0.681 mg, 2.27 mmol) was added. The mixture was stirred at room temperature overnight. The formed salt was removed by filtration and the solution was concentrated. After addition of ethyl acetate (50 mL), the resulting solution was washed with brine. The organic layer was dried with anhydrous magnesium sulfate. After filtration, the solvent was evaporated and the crude product was purified by silica-gel column chromatography (hexane-ethyl acetate = 30:1) to give**1h** as a slightly yellow solid. Yield: 67.7% (1.30 g). Rf: 0.40 (hexane-ethyl acetate = 20:1). IR (KBr): 3700–3100 (OH), 3000–2800 (CH), 1690 (C=O), 1254 (SiC-H), 848 (Si-CH) cm^−1^. ^1^H-NMR (270 MHz, CDCl_3_, ppm) δ: 12.5 (s, 1H, PhOH), 7.97 [s, 1H, HPh(C=O)C], 7.63 [d, 1H, Ph-≡-PhH-O(C=O)], 7.56 (d, 2H, PhOCH_2_PhH), 7.51 (s, 2H, Br-PhH), 7.39 (d, 2H, PhOCH_2_-PhH≡Ph), 6.99 (d, 1H, HO-PhH), 4.89 (s, 2H, PhOCH_2_Ph), 4.68 [s, 4H, 2(PhCH_2_OSi)], 3.02 [t, 2H, Ph(C=O)CH_2_CH_2_], 1.77 (m, 2H, CH_2_CH_2_CH_2_), 1.27 [m, 16H, (CH_2_)_8_ in dodecanone group], 0.92 [s, 18H, 2(Si(CH_3_)_2_C(CH_3_)_3_)], 0.92 (t, 3H, CH_2_CH_3_), 0.09 [s, 12H, 2(Si(CH_3_)_2_C(CH_3_)_3_)].

#### 3.1.9. Synthesis of 1,3-Bis(*tert*-butyldimethylsilyloxymethyl)-2-{4'-[4''-hydroxy-3''-(1-oxododecyl)phenyl-ethynyl]benzyloxy}-5-(trimethylsilylethynyl)benzene (**1i**)

Trimethylsilylacetylene (0.342 mL, 4.60 mmol) was added with a syringe to a mixture of **1h** (1.30 g, 1.53 mmol), triphenylphosphine (32.1 mg, 0.122 mmol), copper iodide (35.0 mg, 0.184 mmol) and bis(triphenylphosphine)palladium (II) chloride (21.5 mg, 30.6 μmol) in triethylamine (10.2 mL). The mixture was stirred at 60 °C for 2 days. The formed salt was removed by filtration and the solution was concentrated. After addition of ethyl acetate (100 mL), the resulting solution was washed with brine. The organic layer was dried with anhydrous magnesium sulfate. After filtration, the solvent was evaporated and the crude product was purified by silica-gel column chromatography (hexane-ethyl acetate = 30:1) to give **1i** as a slightly viscous liquid. Yield: 68.1% (1.68 g). Rf: 0.39 (hexane-ethyl acetate = 20:1). IR (KBr): 3700–3000 (OH), 3000–2800 (CH), 1690 (C=O), 1254 (SiC-H), 848 (Si-CH) cm^−1^. ^1^H-NMR (270 MHz, CDCl_3_, ppm) δ: 12.5 (s, 1H, PhOH), 7.97 [s, 1H, HPh(C=O)C], 7.64 [d, 1H, Ph-≡-PhH-O(C=O)], 7.57 (d, 2H, PhOCH_2_PhH), 7.51 (s, 2H, Si-≡-PhH), 7.37 (d, 2H, PhOCH_2_-PhH≡Ph), 6.99 (d, 1H, HO-PhH), 4.94 (s, 2H, PhOCH_2_Ph), 4.68 [s, 4H, 2(PhCH_2_OSi)], 3.02 [t, 2H, Ph(C=O)CH_2_CH_2_], 1.77 (m, 2H, CH_2_CH_2_CH_2_), 1.27 [m, 16H, (CH_2_)_8_ in dodecanone group], 0.92 [s, 18H, 2(Si(CH_3_)_2_C(CH_3_)_3_)], 0.90 (t, 3H, CH_2_CH_3_), 0.25 [s, 9H, ≡-Si(CH_3_)_3_], 0.08 [s, 12H, 2(Si(CH_3_)_2_C(CH_3_)_3_)].

#### 3.1.10. Synthesis of 1,3-Bis(*tert*-butyldimethylsilyloxymethyl)-2-{4'-[4''-( L-menthoxycarbonyloxy)-3''-(1-oxododecyl)phenylethynyl]benzyloxy}-5-(trimethylsilylethynyl)benzene (**1j**)

Triethylamine (41.8 μL, 0.300 mmol) was added to a solution of **1i** (1.68 g, 1.94 mmol) in THF at room temperature. (−)-menthyl chloroformate (52.6 μL, 0.248 mmol) was added to the solution at room temperature and the solution was stirred for 6 h. After addition of ethyl acetate, the solution was washed with brine and dried with anhydrous sodium sulfate. After filtration, the solvent was evaporated and the crude product was purified by silica-gel column chromatography (hexane-ethyl acetate = 35:1) to give **1j** as a slightly yellow viscous liquid. Yield: 60.3% (1.23 g). Rf: 0.37 (hexane:ethyl acetate = 50:1). IR (KBr): 3000–2800 (CH), 1758 (O(C=O)O), 1690 (C=O), 1256 (SiC-H), 847 (Si-CH) cm^−1^. ^1^H-NMR (270 MHz, CDCl_3_, ppm) δ: 7.90 [s, 1H, HPh(C=O)C], 7.65 [d, 1H, Ph-≡-PhH-O(C=O)], 7.55 (d, 2H, PhOCH_2_PhH), 7.49 (s, 2H, Si-≡-PhH), 7.39 (d, 2H, PhOCH_2_-PhH-≡Ph), 7.21 [d, 1H, HPhO(C=O)O], 4.89 (s, 2H, PhOCH_2_Ph), 4.68 [s, 4H, 2(PhCH_2_OSi)], 4.60–4.50 [m, 1H, PhO(C=O)OCH, in menthoxy group], 2.90 [t, 2H, Ph(C=O)CH_2_CH_2_], 2.22–2.05 (m, 2H, methylene, methinein menthoxy group), 1.69 (m, 4H, CH_2_CH_2_CH_2_ in dodecanone group and methylene, methinein menthoxy group), 1.51 (m, 2H, methylene in menthoxy group), 1.26 (m, 16H, (CH_2_)_8 _in dodecanone group), 1.20–1.02 (m, 3H, methylene, methine in menthoxy group), 0.95 [m, 6H, 2(CH_3_CH) in menthoxy group], 0.92 [s, 18H, 2(Si(CH_3_)_2_C(CH_3_)_3_)], 0.87 (m, 6H, CH_2_CH_3 _in dodecanone and CH_3_CH in menthoxy group), 0.26 [s, 9H, ≡-Si(CH_3_)_3_], 0.08 [s, 12H, 2(Si(CH_3_)_2_C(CH_3_)_3_)].

#### 3.1.11. Synthesis of 3,5-Bis(hydroxymethyl)-4-{4'-[4''-(L-menthoxycarbonyloxy)-3''-(1-oxododecyl)-phenylethynyl]benzyloxy}phenylacetylene (**1**)

A mixture of tetrabutylammonium fluoride (1 M in THF) (0.20 mL, 0.20 mmol) and acetic acid (0.20 mL) (=1/1 (v/v)) was added to a solution of **1j** (152 mg, 0.145 mmol) in THF (0.51 mL). The solution was stirred at room temperature for 12 h. After addition of ethyl acetate, the solution was washed with brine and dried with anhydrous sodium sulfate. After filtration, the solvent was evaporated and the crude product was purified by silica-gel column chromatography (hexane-ethyl acetate = 3:1) to give **1** as a white solid. Yield: 21.8% (49.1 mg). Rf: 0.16 (hexane-ethyl acetate = 3:1). IR (KBr): 3400–3000 (-OH), 3296 (≡C-H), 2954–2854 (C-H), 1759 [O(C=O)O], 1692 (C=O). [α]_D_^20^ −18.39 °(*c* 0.500, THF). ^1^H-NMR (270 MHz, CDCl_3_, ppm) δ: 7.90 [s, 1H, HPh(C=O)C], 7.64 [d, 1H, Ph-≡-PhH-O(C=O)], 7.56 (d, 2H, PhOCH_2_PhH), 7.52 (s, 2H, ≡-PhH), 7.41 (d, 2H, PhOCH_2_-PhH-≡-Ph), 7.20 [d, 1H, HPhO(C=O)O], 4.97 (s, 2H, PhOCH_2_Ph), 4.66 [s, 4H, 2(PhCH_2_OH)], 4.59–4.46 [m, 1H, PhO(C=O)OCH in menthoxy group], 3.06 (s, 1H, HC≡C), 2.90 [t, 2H, Ph(C=O)CH_2_CH_2_], 2.21–1.80 (m, 2H, methylene, methine, in menthoxy group), 1.70 (m, 4H, CH_2_CH_2_CH_2_ in dodecanone group and methylene, methine in menthoxy group), 1.51 (m, 2H, methylene in menthoxy group), 1.26 [m, 16H, (CH_2_)_8 _in dodecanone group], 1.19–1.02 (m, 3H, methylene, methinein menthoxy group), 0.94 [br, 6H, 2(CH_3_CH) in menthoxy group], 0.87 (m, 6H, CH_2_CH_3_ in dodecanone and CH_3_CH in menthoxy group). ^13^C-NMR (CDCl_3_, ppm) δ:199.5 (C=O), 154.9 (Ph), 152.5 (Ph), 148.7 (C=O), 136.9 (Ph), 135.5 (Ph), 134.5 (Ph), 132.8 (Ph), 132.6 (Ph), 131.9 (Ph), 131.4 (Ph), 127.9 (Ph), 123.5 (Ph), 122.8 (Ph), 121.3 (Ph), 118.6 (Ph), 90.0 (C≡C), 88.2 (C≡C), 83.0 (C≡C), 80.2 (C≡C), 77.2 (CH_2_OPh), 76.5 [O-CH(C-) (C-)], 60.5 (-C-OH), 34.1, 29.7, 29.5, 29.6, 29.4, 29.3, 26.2, 22.8, 14.2 (dodecanone group), 47.0, 40.6, 34.1, 32.0, 26.2, 23.4, 22.0, 20.8, 16.4 (menthoxy group). Anal. Calcd for C_48_H_60_O_7_: C, 76.97; H, 8.07. Found. C, 76.87; H, 7.92.

### 3.2. Asymmetric-Induced Polymerization (AIP) and Polymer Reaction in Membrane State (RIM) (see Scheme 1 and Scheme 2)

#### 3.2.1. Asymmetric-Induced Polymerization (**AIP**) of **1**

A solution of triethylamine (34.5 μL, 0.248 mmol) and [Rh(nbd)Cl]_2_ (0.572 mg, 1.24 μmol) in toluene (0.263 mL) was added with a syringe to a solution of **1** (92.9 mg, 0.124 mmol) in toluene (0.265 mL). After the solution was stirred at room temperature for 6 h, the solution was diluted with toluene (30 mL). The solution was poured into a large amount of methanol to precipitate a red solid, which was filtered out and dried *in vacuo*. poly(**1**): Yield 98.6% (91.6 mg). [α]_D_^20^ −176° (*c* 0.100, THF). IR (film): 3750–3100 (-OH), 2954–2854 (C-H), 1761 [O(C=O)O], 1696 (C=O). ^1^H-NMR (270 MHz, CDCl_3_, ppm) δ: 7.80–7.11 (br, 9H, PhH), 5.91 (br, 0.7H, CH_2_=CH), 4.83 (br, 2H, PhOCH_2_Ph), 4.55 [br, 5H, 2 (PhCH_2_OH) and PhO(C=O)OCH in menthoxy group], 2.82 [br, 2H, Ph(C=O)CH_2_CH_2_], 2.13 (br, 2H, methylene, methine in menthoxy group), 1.72 (br, 2H, CH_2_CH_2_CH_2_), 1.62 (br, 2H, methylene, methine in menthoxy group), 1.48 (br, 2H, methylene in menthoxy group), 1.25 [br, 16H, (CH_2_)_8_ in dodecanone group], 1.20–1.00 (br, 3H, methylene, methine in menthoxy group), 0.94 [br, 6H, 2(CH_3_CH) in menthoxy group], 0.87 (br, 6H, CH_2_CH_3_, in dodecanone group and CH_3_CH in menthoxy group).

#### 3.2.2. Synthesis of Poly(***de*-1**) by Desubstitution of the Chiral Groups from Poly(1) in Membrane State (**RIM**)

A solution of poly(**1**) in chloroform ((1.5 ml, 30 mg/mL) was cast on a poly(tetrafluoroethylene) sheet (40 cm^2^), and the solvent was evaporated for 24 h at room temperature. The resulting free-standing membrane was detached from the sheet and dried *in vacuo* for 24 h. The membrane was immersed in a solution of potassium hydroxide in THF/methanol (volume ratio 1/3) to desubstitute the chiral groups at room temperature for 36 h. After the reaction, the membrane was washed with methanol and then to neutralize it was immersed in a mixture of methanol/acetic acid (volume ratio 4/1) at room temperature for 1 h. The membrane was immersed in methanol at room temperature overnight to remove the chiral groups remained. After washing the membrane with methanol, the membrane was dried in vacuo overnight. poly(***de*-1**): The weight loss (%), calcd: 24.28, found: 24.21. IR (film): 3750–3100 (-OH), 2925–2855 (C-H), 1693 (C=O).

#### 3.3.3. Synthesis of Poly(re-1) by Resubstitution of the Achiral Groups to poly(de-1) in Membrane State (**RIM**)

The membrane of poly(***de*-1**) was immersed in lauroyl chloride at 60 °C for 30 min. After the reaction, the membrane was washed with water, methanol and then hexane. The membrane was immersed in methanol for 6 h to remove the chiral groups remained. After immersing the membrane in hexane overnight, the resulting membrane was dried in vacuo for 24 h. poly(***re*-1**): Yield 91.4%. [α]_D_^20^ 117° (*c* 0.100, THF). IR (film): 3750–3100 (-OH), 2926–2857 (C-H), 1752 [O(C=O)], 1694 (C=O). ^1^H-NMR (270 MHz, CDCl_3_, ppm) δ:7.80–6.73 (br, 9H, PhH), 5.83 (br, 0.6H, CH_2_=CH), 4.87 [br, 6H, 2(PhCH_2_OH) and PhOCH_2_Ph,)], 2.85 [br, 2H, Ph(C=O)CH_2_CH_2_], 2.34 [br, 2H, PhO(C=O)CH_2_CH_2_], 1.68 [br, 4H, 2(CH_2_CH_2_CH_2_) in dodecanone groups], 1.24–1.13 [br, 32H, 2(CH_2_)_8_ in dodecanone groups], 0.87 [br, 3H, Ph(C=O)(CH_2_)_10_CH_3_], 0.81 [br, 3H, PhO(C=O)(CH_2_)_10_ CH_3_].

#### 3.3.4. Synthesis of Poly(***de-2***) by Removing the Chiral Groups from Poly(2) in Membrane State (**RIM**) (Scheme 3)

According to a procedure similar to that in 3.2.1 and 3.2.2, poly(**2**) and its membrane were prepared. The membrane was immersed in methanol/2N hydrochloric acid (volume ratio 1/1) at 0 *°*C for 3 days. It was neutralized in 28 wt% aqueous ammonia for 12 h, washed with methanol, and dried *in vacuo* for 24 h. poly(*de*-2): The weight loss (%), calcd: 29.69, found: 29.58. IR (film): 3750–3100 (-OH), 2950–2855 (C-H).

## 4. Conclusions

A soluble and stable one-handed helical poly(substituted phenylacetylene) without the coexistence of any other chiral moieties was successfully synthesized by **AIP** of a chiral monomer having a chiral group, followed by two-step **RIM**s of the resulting polymer: removing the chiral groups *(desubstitution)*, and introduction of achiral long alkyl groups at the same position to enhance the solubility of the resulting one-handed helical polymer *(resubstitution)*. There were two possible reasons for the success: (1) the one-handed helical main chain was maintained because the two polymer reactions were carried out in membrane state; and (2) the polymer’s solubilities were maintained by introduction of the two suitable hydrophobic groups to proper positions in the starting monomer. The one-handed helical main chain was kept due to stabilization by intramolecular hydrogen bonds. Moreover, the difference in g values between the starting and the final polymer was small.
